# Etiology and Mortality of Nonconvulsive Status Epilepticus

**DOI:** 10.3390/neurolint17020029

**Published:** 2025-02-17

**Authors:** Firdevs Ezgi Uçan Tokuç, Emine Görgülü, Fatma Genç, Meltem Korucuk, Abidin Erdal, Yasemin Biçer Gömceli

**Affiliations:** 1 Department of Neurology, University of Health Sciences, Antalya Training and Research Hospital, Antalya 07100, Turkey; gorguluamine@gmail.com (E.G.); sanivardr@yahoo.com (F.G.); meltem_yasar@yahoo.com (M.K.); 2 Department of Neurology, Ankara Bilkent City Hospital, Health Ministry of Turkiye Republic, Ankara 06800, Turkey; abidinerdal@gmail.com; 3 Department of Neurology, Memorial Antalya Hospital, Antalya 07025, Turkey; yasemingomceli@hotmail.com

**Keywords:** epilepsy, nonconvulsive status epilepticus, etiology, mortality, antiseizure medication

## Abstract

Objectives: Nonconvulsive status epilepticus (NCSE) is a disease with a high mortality rate and a very diverse etiology. The disease prognosis is related to the etiology. We aimed to investigate the etiology, mortality rates, and factors affecting mortality in patients diagnosed with NCSE in a tertiary epilepsy center in Turkey. Methods: All electroencephalograms (EEGs) were taken in the EEG laboratory of the Department of Neurology, Antalya Training and Research Hospital, between June 2021 and February 2024. Patients who met the Salzburg Consensus Criteria (SCC) for NCSE were included. Demographic data, etiologic factors, comorbidities, neuroradiological imaging, laboratory data, treatments administered for NCSE and responses to treatment, short- and long-term outcomes, and EEG findings at follow-up, if any, were noted from the medical records of all patients who met the criteria. Results: A total of 200 patients were included in the study. Mortality was observed in 76 (38.4%) patients with NCSE. There was a statistically significant correlation between NCSE etiology and mortality (*p* < 0.001). Mortality was most common in patients with cerebral tumors as the etiology, with a rate of 63.6%. The lowest mortality rate was observed in patients with autoimmune encephalitis and epilepsy (14.3% and 17.2%, respectively). After appropriate antiseizure medication (ASM) treatment, 117 (58.5%) patients with NCSE improved. When post-treatment improvement and etiologic factors were analyzed, the highest rate of improvement was observed in the autoimmune encephalitis and CVD groups. Conclusions: Our study showed that advanced age and the presence of stroke are associated with mortality and that patients with NCSE due to autoimmune encephalitis respond well to treatment.

## 1. Introduction

Nonconvulsive status epilepticus (NCSE) is an epileptic state in which major convulsive movements are not observed, but an altered or decreased level of consciousness, or vegetative or subjective behavioral symptoms are observed [1]. Although it is not rare, it is very challenging to determine its prevalence. It is considered to be more frequent than reported since it is usually an under-recognized diagnosis and may be confused with metabolic encephalopathies. This condition causes a delay in treatment [2]. In previous studies conducted on patients with altered mental status, publications that reported a prevalence of NCSE ranging between 5% and 48% are available [1]. In a similar prospective study conducted in Qatar using continuous electroencephalograms (cEEG), this rate was observed to be 26% [3]. Therefore, recognition of the disease and electroencephalograms (EEG) are critical for diagnosis.

Although it is observed frequently in patients with a previous diagnosis of epilepsy, it is observed more frequently in elderly and critically ill patients [4]. The potential for NCSE to cause brain damage and its mortality and morbidity rates are still highly controversial [5]. Some studies have reported high morbidity and mortality, while others have described it as a benign disease that does not require aggressive treatment [6,7]. In this study, we aimed to investigate the etiology, mortality rates, and factors affecting mortality in patients diagnosed with NCSE in a tertiary epilepsy center in Turkey.

## 2. Materials and Methods

### 2.1. Patient Population and Data Collection

All EEGs taken in the EEG laboratory of the Department of Neurology, Antalya Training and Research Hospital, between June 2021 and February 2024 were screened. EEG findings were analyzed by 2 experienced epileptologists. Patients who met the Salzburg Consensus Criteria (SCC) for NCSE were included [8]. Demographic data, etiological factors, seizure history, comorbidities, neuroradiological imaging, laboratory data, treatments for NCSE and response to treatment, short- and long-term outcomes, and follow-up EEG findings, if any, were recorded from the medical records of all patients who met the criteria. The applied exclusion criteria were as follows: EEG abnormalities that did not meet the SCC, patients who could not be followed up on, or patients who did not receive treatment were not included in the study. The study was approved by the Ethics Committee of the Antalya Training and Research Hospital (2024–071).

### 2.2. Diagnosis of NCSE

Twenty-one-channel EEG recordings, with additional anterior temporal electrodes (T1 and T2) placed according to the international 10–20 system for at least a 30 min duration, were recorded from each patient. Some EEG recordings included video recordings. The EEG technician noted all clinical findings during the recording and the clinical response to diazepam, if administered. The EEG findings were categorized according to the American Clinical Neurophysiology Society (ACNS)’s standardized critical EEG terminology [9]. In the EEG, background EEG activity (frequency, symmetry, and reactivity), and the localization, morphology, frequency changes, amplitude, and spatial spreading of ictal patterns, as well as the response to intravenous diazepam, if administered, were noted. The diagnosis of NCSE was classified as ‘definite NCSE’, ‘possible NCSE’, or ‘no NCSE’ according to the SCC [8].

In addition, the etiology of NCSE was grouped as acute, remote, or progressive symptomatic according to the International League Against Epilepsy (ILAE) classification [10]. According to the response to treatment, status epilepticus (SE) was classified as responsive or refractory. Response to treatment was defined as the cessation of SE after first-line treatment with benzodiazepines, followed by second-line treatment with an intravenous antiseizure medication (ASM). Refractory SE (RSE) was defined as a failure of first-line therapy with benzodiazepines and one second-line treatment with ASMs [11]. Anesthetics such as propofol, ketamine, and thiopental, which are used in the treatment of refractory status epilepticus, were not used for treatment in any patient. EEGs were grouped according to the improvement in the ictal activity in EEGs taken after second-line treatment. Patients who could not have an EEG or who died before an EEG could be taken were included in the ’no control EEG’ group.

### 2.3. Statistical Analysis

The data were analyzed with IBM SPSS V23. Compliance with the normal distribution was examined by the Kolmogorov–Smirnov test. The chi-square test, Yates correction, and Fisher–Freeman–Halton tests were used to compare categorical variables according to their groups. The Mann–Whitney U test was used to compare non-normally distributed data according to binary groups. Binary logistic regression analysis was utilized to examine the risk factors for mortality and deterioration. The significance level was taken as *p* < 0.050.

## 3. Results

A total of 200 patients were included in the study. Of the patients, 111 (55.5%) were female and 89 (44.5%) were male, with a mean age of 58.84 ± 21.47 years (age range 18–93 years). A total of 12 patients were using ASM with other diagnoses (bipolar disorder, trigeminal neuralgia), although they had not had seizures before. A total of 80 (40%) patients had a history of epilepsy, and 67 (30.2%) of these patients had epilepsy as the etiology of NCSE. The second most common etiology of NCSE was cerebrovascular disease (CVD), with a frequency of 39 (17.6%). The demographic data of the patients are summarized in Table 1.

Of the 80 patients with a previous diagnosis of epilepsy, 7 were in the generalized epilepsy group, 3 in the unclassifiable group, and 70 in the focal epilepsy group.

Mortality was observed in 76 (38.4%) patients with NCSE, and no survival or mortality information was available for 2 patients. There was a statistically significant correlation between the etiology of NCSE and mortality (*p* < 0.001). Mortality was most common in patients with brain tumors, with a rate of 63.6%, followed by a rate of 57.1% in patients with dementia and 53.8% in patients with CVD. The lowest mortality rates were observed in patients with autoimmune encephalitis and epilepsy (14.3% and 17.2%, respectively) (Table 2).

Furthermore, when the etiologies were classified as acute, remote, and progressive symptomatic, and the relationship with mortality was analyzed, no statistically significant difference was observed (*p* = 0.166).

When the risk factors affecting mortality were analyzed univariately, the mortality risk increased 1.061-fold with increasing age (*p* < 0.001). The mortality risk was lower in patients with epilepsy than in those without epilepsy (OR = 0.270; *p* < 0.001). The risk of mortality was lower in those who used ASMs than in those who did not (OR = 0.441; *p* = 0.007). The mortality risk of those receiving polytherapy was lower than that of those receiving monotherapy (*p* = 0.007) (Table 3 and Table 4).

When multiple models were analyzed, the mortality risk increased 1.064-fold with increasing age (*p* < 0.001). Those with improvements in their EEGs after treatment had a lower mortality risk (*p* = 0.028). Other variables were not identified as risk factors for mortality (*p* > 0.050). The mortality risk was 2530 times greater in those with CVD as the etiology than in those without (Table 3 and Table 4).

After appropriate ASM treatment, 117 (58.5%) patients with NCSE improved, while no change was observed in 19 (9.5%) patients. A deterioration of the EEG was observed in 12 (26%) patients. In 52 patients, no control EEG was observed due to death, discharge, etc. When post-treatment improvement and etiological factors were analyzed, the highest rate of improvement was observed in the autoimmune encephalitis and CVD groups (Table 5).

When the relationship between post-treatment improvement and other independent variables was examined, the correlation between the deterioration in EEGs and polytherapy/monotherapy was statistically significant (*p* = 0.034). The rate of deterioration was 64.1% in those receiving monotherapy and 89.7% in those receiving polytherapy. No significant correlation was found between the other variables (*p* > 0.050). When the risk factors affecting the post-treatment EEG were analyzed univariately, the risk of deterioration of the post-treatment EEG result was found to be lower in those who were receiving polytherapy than in those who were receiving monotherapy (OR= 0.206; *p* = 0.023). When multiple models were analyzed, the risk of the EEG results worsening after treatment was lower in those with epilepsy than in those without epilepsy (OR =0.050; *p* = 0.031). The risk of post-treatment EEG deterioration was higher in those who used ASMs than in those who did not (*p* = 0.024). Other variables were not found to be risk factors for post-treatment EEG deterioration (*p* > 0.050) (Table 6 and Table 7).

## 4. Discussion

In this single-center retrospective study, we investigated the etiology and factors influencing mortality in patients diagnosed with NCSE. NCSE is a disease with a high mortality rate, which can reach 50–52%, especially in critically ill patients [12,13]. In our study, the mortality rate was 38.4%. We believe that our relatively low mortality rate may be due to the high number of young patients and the fact that the patient group included in the study did not consist only of intensive care unit patients.

The etiology of NCSE is one of the most debated issues affecting prognosis. Some studies have reported that the prognosis is worse in patients with NCSE caused by acute pathologies, while others have reported that acute and chronic etiologies do not contribute to mortality [6,14,15,16]. In our study, there was no association between the type of etiology (acute, remote, or progressive) and mortality. However, in univariate analyses, mortality was higher in patients with a history of cerebrovascular disease than in those without. In fact, this effect is twofold. In a study by Kitchener et al., the presence of NCSE in patients with stroke was associated with a higher mortality. In another study, the presence of CVD was associated with a higher mortality in patients with NCSE [3,17]. Our results are consistent with this finding.

In addition, in our study, advanced age and no previous history of epilepsy were associated with mortality. Previous studies have shown that although the rate of diagnosis of NCSE decreases in the elderly population, mortality in elderly individuals is higher than that in younger individuals (31% vs. 7%) [13,18]. Additionally, although many studies have associated the absence of a previous diagnosis of epilepsy with poor prognosis and high mortality, the reason for this association has not been fully elucidated. However, it has been discussed that this may suggest an association between de novo SE and severe acute structural brain injury leading to poor outcomes [16,19].

One of the important findings of our study is that the etiology of NCSE can be quite diverse [19]. Another conclusion is that patients with autoimmune encephalitis in the etiology of NCSE may have a good prognosis, and all patients in our study responded well to treatment. Although autoimmune encephalitis did not reach statistical significance due to the small number of patients, this finding raises the question of whether autoimmune encephalitis in the etiology of NCSE may be a criterion for a good prognosis. Considering that immunotherapeutic agents used to treat autoimmune encephalitis are also used to treat RSE, we believe that the treatment administered may have contributed to the prognosis [20]. Autoimmune encephalitis has been recognized relatively recently, and in most less developed or developing countries there are diagnostic deficiencies due to difficulties in antibody testing and an under-recognition of the diagnosis. Therefore, most studies have not included autoimmune encephalitis among the etiologies of NCSE. As a result, there is no large-scale study in the literature on autoimmune encephalitis and prognosis in the etiology of NCSE. However, a study by Mitchell et al. in 2020 reported that autoimmune encephalitis may be as common as epilepsy in the etiology of NCSE [21]. Although it is known that seizures may be refractory in patients with autoimmune encephalitis, it is noteworthy that the prognosis in patients with autoimmune encephalitis is not poor according to a few studies that also investigated autoimmune encephalitis in the etiology [22,23,24]. Our study highlights the need for large-scale studies to develop this thesis, which has not been emphasized so far. However, studies on how to make aggressive treatment decisions for NCSE are still controversial. Despite this, recent guidelines recommend that patients with NCSE should be treated similarly to patients with convulsive SE [23,24]. Although the choice of agent and the duration of treatment should be individualized for each patient, retrospective clinical series have shown that first-line intravenous treatment includes benzodiazepines, levetiracetam, valproate, fosphenytoin, lacosamide, brivaracetam, or phenobarbital, with response rates ranging from 53% to 90% [24,25]. In our study, the response rate to treatment was 58.5%, which is consistent with the literature.

In a previous study conducted in Qatar, 54.5% remission was achieved after NCSE treatment [3]. Refractory NCSE rates have been reported to vary between 10 and 20% [26]. In our study, the remission rates were also similar to those reported in the literature and the refractory NCSE rates were slightly lower. The reason for this is that 26% of patients did not have a post-treatment EEG. We believe that this is related to the low rates of refractory NCSE.

Our study has several limitations. It is retrospective, and some data could not be obtained. In addition, it was a single-center study and had a relatively small sample size when the data were divided into etiological subcategories. Because we did not use cEEG, we may have underestimated the frequency of NCSE, especially in comatose patients admitted to intensive care, which may have led us to misjudge the treatment response in these patients.

## 5. Conclusions

In conclusion, although the etiology of NCSE can be quite diverse, it is a disease with a high mortality rate. Etiology is a factor that influences both mortality and response to treatment. Our study showed that advanced age and the presence of stroke are associated with mortality. Furthermore, all NCSE patients with autoimmune encephalitis as the etiology responded well to treatment, suggesting that patients with NCSE due to autoimmune encephalitis may respond well to treatment. Multicenter, large-scale, and prospective studies will be useful in shedding light on this issue.

## Figures and Tables

**Table 1:** Results of frequency analysis of variables.

	Frequency	Percentage
Sex		
Female	111	55.5
Male	89	44.5
Previous Epilepsy Diagnosis		
Yes	80	40.0
No	120	60.0
Previous use of ASMs		
Yes	92	46
No	108	54
Polytherapy/Monotherapy		
Monotherapy	53	57.6
Polytherapy	39	42.4
Epilepsy classification		
Focal Epilepsy	190	95.0
Generalized epilepsy	7	3.5
Unclassified	3	1.5
Previous NCSE		
Yes	58	29.0
No	95	47.5
Unknown	47	23.5
Etiology *		
Stroke	39	17.6
CNS infection	12	5.4
Cerebral tumor	23	10.4
Epilepsy	67	30.2
Dementia	7	3.2
Intracranial hemorrhage	19	8.6
Electrolyte imbalance	15	6.8
Systemic infection	28	12.6
Autoimmune encephalitis	7	3.2
Hypoxic-ischemic encephalopathy	5	2.3
Etiology (by category)		
Acute symptomatic	88	44
Remote symptomatic	26	13
Progressive symptomatic	86	43
Mortality		
Yes	76	38.4
No	122	61.6

* Indicates multiple responses. CNS: central nerve system, ASMs: antiseizure medications, and NCSE: nonconvulsive status epilepticus.

**Table 2:** Analyzing the relationship between etiology and mortality.

	Mortality		
	Mortality	No Mortality	
	*n* (%)	*n* (%)	*p*
Etiology			
CVD	21 (53.8)	18 (46.2)	0.041 (**)
CNS infection	4 (33.3)	8 (66.7)	0.660 (**)
Mass in brain	14 (63.6)	8 (36.4)	0.014 (*)
Epilepsy	12 (17.9)	55 (82.1)	<0.001 (*)
Dementia	4 (57.1)	3 (42.9)	0.327 (**)
Intracranial hemorrhage	7 (36.8)	12 (63.2)	0.813 (*)
Electrolyte imbalance	7 (50)	7 (50)	0.548 (*)
Infection and others	16 (57.1)	12 (42.9)	0.066 (*)
Autoimmune encephalitis	1 (14.3)	6 (85.7)	0.248 (**)
HIE	1 (20)	4 (80)	0.552 (**)
Test statistics	39.012		
*p* *	<0.001		

* Pearson’s chi-square test; ** Fisher’s exact test. CVD: cerebrovascular disease, CNS: central nervous system, and HIE: hypoxic-ischemic encephalopathy.

**Table 3:** Logistic regression analysis of risk factors affecting mortality.

	Mortality		Univariate		Multiple	
	No	Yes	OR (%95 CI)	*p*	OR (%95 CI)	*p*
Age	50.8 ± 21.3	71.4 ± 14.6	1.061 (1.04–1.082)	<0.001	1.064 (1.034–1.094)	<0.001
Sex						
Female	65 (59.1)	45 (40.9)	Reference			
Male	57 (64.8)	31 (35.2)	1.273 (0.713–2.272)	0.414	1.003 (0.511–1.969)	0.991
Epilepsy						
No	59 (50)	59 (50)	Reference			
Yes	63 (78.8)	17 (21.3)	0.27 (0.141–0.515)	<0.001	0.373 (0.064–2.187)	0.275
Previous ASM						
No	56 (52.8)	50 (47.2)	Reference			
Yes	66 (71.7)	26 (28.3)	0.441 (0.244–0.798)	0.007	2.077 (0.659–6.544)	0.212
ASM						
Monotherapy	32 (60.4)	21 (39.6)	Reference			
Polytherapy	34 (87.2)	5 (12.8)	0.224 (0.075–0.665)	0.007	--	--

ASM: antiseizure medication.

**Table 4:** Examination of risk factors affecting mortality with logistic regression analysis (continued).

	Mortality		Univariate	*p*	Multiple	*p*
	No	Yes	OR (%95 CI)		OR (%95 CI)	
Post-treatment EEG						
Not availiable	31(59.6)	21(40.4)	Reference			
Improved	77(66.4)	39(33.6)	0.748 (0.381−1.468)	0.398	0.347 (0.135−0.891)	0.028
No change	9 (50)	9(50)	1.476 (0.503−4.335)	0.479	0.785 (0.125−4.915)	0.796
Deteriorated	5 (41.7)	7 (58.3)	2.067 (0.578−7.391)	0.264	0.937 (0.155−5.666)	0.944
Etiology CVD No	106(65.8)	55(34.2)		Reference		
Yes	16(43.2)	21(56.8)	2.53 (1.222–5.235)	0.012	1.798 (0.583–5.549)	0.307

CVD: cerebrovascular disease; and EEG: electroencephalograms.

**Table 5:** Examination of the relationship between etiology and post-treatment EEG.

	Post-Treatment EEG			
	Improved	No Change	Deteriorated	No Control EEG
	*n* (%)	*n* (%)	*n* (%)	*n* (%)
Etiology				
CVD	29 (74.4)	3 (7.7)	1 (2.6)	6 (15.4)
CNS infection	7 (58.3)	1 (8.3)	--	4 (33.3)
Cerebral tumor	9 (39.1)	9 (13)	2 (8.7)	9 (39.1)
Epilepsy	39 (58.2)	6 (9)	3 (4.5)	19 (28.4)
Dementia	3 (42.9)	--	1 (14.3)	3 (42.9)
Intracranial hemorrhage	9 (47.4)	4 (21.1)	1 (5.3)	5 (26.3)
Electrolyte imbalance	8 (53.3)	1 (6.7)	1 (6.7)	5 (33.3)
Infection and other	13 (46.4)	2 (7.1)	4 (14.3)	9 (32.1)
Autoimmune encephalitis	7 (100)	--	--	--
HIE	2 (40)	--	--	3 (60)
Test statistics	31.213			
*p* *	0.405			

* Pearson’s chi-square test. CVD: cerebrovascular disease, CNS: central nervous system, and HIE: hypoxic-ischemic encephalopathy.

**Table 6:** Logistic regression analysis of risk factors affecting post-treatment EEG.

	Post-Treatment EEG		Univariate	Multiple		
	Improved	Deterioration-No Change	OR (%95 CI)	*p*	OR (%95 CI)	*p*
Age	59.3 ± 20.9	64.2 ± 21.9	1.012 (0.992–1.032)	0.256	1.012 (0.98–1.044)	0.472
Sex						
Female	65 (79.3)	17 (20.7)	Reference			
Male	52 (78.8)	14 (21.2)	1.029 (0.464–2.281)	0.943	1.097 (0.481–2.502)	0.824
Epilepsy						
No	69 (78.4)	19 (21.6)	Reference			
Yes	48 (80)	12 (20)	0.908 (0.403–2.043)	0.815	0.05 (0.003–0.763)	0.031
Previous ASM						
No	64 (82.1)	14 (17.9)	Reference			
Yes	51 (75)	17 (25)	0.656 (0.296–1.457)	0.300	0.211 (0.054–0.818)	0.024
ASM						
Monotherapy	25 (64.1)	14 (35.9)	Reference			
Polytherapy	26 (89.7)	3 (10.3)	0.206 (0.053–0.805)	**0.023**	---	---
Previous NCSE						
Unknown	26 (83.9)	5 (16.1)	Reference			
Yes	34 (70.8)	14 (29.2)	2.141 (0.684–6.706)	0.191	2.89 (0.721–11.614)	0.134
No	57 (82.6)	12 (17.4)	1.095 (0.35–3.429)	0.877	0.189 (0.025–1.407)	0.104

ASMs: antiseizure medications, NCSE: nonconvulsive status epilepticus.

**Table 7:** Examination of risk factors affecting post-treatment EEG with logistic regression analysis (continued).

	Post-Treatment EEG		Univariate		Multiple	
	Improved	Deterioration-No Change	OR (%95 CI)	*p*	OR (%95 CI)	*p*
Etiology CVD						
No	90 (76.9)	27 (23.1)	Reference			
Yes	27 (87.1)	4 (12.9)	0.494 (0.159–1.536)	0.223	1.011 (0.15–6.803)	0.991
MRI findings						
MRI normal	40 (75.5)	13 (24.5)	Reference			
Lesion in MRI	77 (81.1)	18 (18.9)	0.719 (0.32–1.616)	0.425	0.682 (0.203–2.289)	0.536
Mortality						
No	77 (84.6)	14 (15.4)	Reference			
Yes	39 (70.9)	16 (29.1)	2.256 (1–5.093)	0.050	2.61 (0.622–10.979)	0.190

MRI: magnetic resonance imaging, CVD: cerebrovascular disease.

## Data Availability

The original contributions presented in this study are included in this article; further inquiries can be directed to the corresponding author.
